# The Vegetative State – A Syndrome in Search of a Name

**Published:** 2012-03-05

**Authors:** K von Wild, ST Laureys, F Gerstenbrand, G Dolce, G Onose

**Affiliations:** * Medical Faculty University Muenster, Cerebprotect Foerderverein für Frührehabilitation e.V., 48155 Muenster, Germany; ** Department of Neurology Liège University Hospital & Belgian National Funds for Scientific Research Cyclotron Research Centre University of Liège, Liège, Belgium; *** Research Institute for Space-Neurology and Neurorehabilitation. 1090 Vienna, Austria; **** Istituto S. Anna Di Ezio Pugliese S.R.L, Crotone, Italy; ***** "Carol Davila" University of Medicine and Pharmacy & ”Bagdasar-Arseni” Teaching Emergency Hospital, Bucharest, Romania

**Keywords:** Apallic syndrome, unresponsive wakefulness syndrome, severe brain damage, neurobehavioral disturbances

## Abstract

In 2002, Bryan Jennett chose the caption “A syndrome in search of a name” for the first chapter of his book “The vegetative state - medical facts, ethical and legal dilemmas”, which, in summary, can be taken as his legacy. Jennett coined the term "VegetativeState" (VS), which became the preferential name for the syndrome of wakeful unresponsiveness in the English literature, with the intention to specify the concern and dilemmas in connection with the naming "vegetative", "persistent" and "permanent". In Europe, Apallic Syndrome (AS) is still in use. The prevalence of VS/AS in hospital settings in Europe is 0.5–2/100.000 population year; one-third traumatic brain damage, 70% following intracranial haemorrhages, tumours, cerebral hypoxemia after cardiac arrest, and end stage of certain progressive neurological diseases. VS/AS reflects brain pathology of (a) consciousness, self-awareness, (b) behaviour, and (c) certain brain structures, so that patients are awake but total unresponsive. The ambiguity of the naming “vegetative” (meant to refer to the preserved vegetative (autonomous nervous system) can suggest that the patient is no more a human but “vegetable” like. And “apallic” does not mean being definitively and completely anatomically disconnected from neocortical structures. In 2009, having joined the International Task Force on the Vegetative State, we proposed the new term “Unresponsive Wakefulness Syndrome” (UWS) to enable (neuro-)scientists, the medical community, and the public to assess and define all stages accurately in a human way. The Unresponsive Wakefulness Syndrome (UWS) could replace the VS/AS nomenclature in science and public with social competence.

## Introduction

Replacing the terms "PersistentVegetativeState" (PVS) and "Apallic Syndrome" (AS) for this characteristic clinical syndrome appeared to be impossible without introducing another, more appropriate name. Based on many years’ experience and the literature, the term "Unresponsive Wakefulness Syndrome" (UWS) seems suitable to replace the controversial issue of VS and AS nomenclature. Recent functional fMRI and PET studies have brought forward new arguments corroborating that alterations of the state of awareness/consciousness are difficult to be systematized and subsequently denominated, and this has been one of the reasons for more experimental clinical studies on this matter - as it is the case with this new article.

## Materials and methods

A steadily increasing number of patients, who would have died in the past, survive severest traumatic and non-traumatic brain damage thanks to modern emergency and intensive care treatment, diagnostic neuroimaging, and long-term caring [**1**]. This, however, could be at the expense of long-lasting and even permanent severe impairments of higher brain functioning [**2-6**], causing specific signs and symptoms known in continental Europe, Asia, and Japan as *Apallic Syndrome* (AS) [**3,7,8**] while in the English speaking countries and the literature, the preferred term is (persistent/permanent) *Vegetative State* ((P)VS) [**9-12**]. The correct diagnosis and management of patients suffering from VS/AS have been the subject of sustained scientific and moral-legal debates over the past decades [**5,11,13,14**]. A growing number of physicians, healthcare workers, politicians, related and religious groups and lay-press journalists’ commentaries have felt uncomfortable when referring to patients as vegetative [**5,11,14-16**]. New insight into VS/AS following the study of brain functions by neuroimaging techniques led to rejecting the concept of “A-pallic” in the sense of complete disconnection of the neocortical structures. Having this in mind, as well as the negative associations intrinsic to the term “vegetative state” and the diagnostic errors with their potential effect on the treatment (potential withdrawal of fluid and nutrition), experts of clinical care recently proposed to replace the terms VS and AS by the new and correct terminology *Unresponsive Wakefulness Syndrome (UWS)* [**15**].

The overall incidence of new AS/VS full stage cases of all aetiology is reported in the literature to be 0.5 - 2/ 100.000 population per year. About one third is traumatic and two thirds are non-traumatic cases. Increasing figures are noticed for hypoxic brain damage and progressive neurological disease (i.e. Mb. Alzheimer, Parkinson’s disease) [**3,7,9**]. The main conceptual criticism is based on the assessment and diagnosis of all different AS/VS stages based solely on *behavioural findings* without knowing the exact or uniform pathogenesis, neuropathologic findings, and the uncertainty of clinical assessment due to varying inclusion criteria [**2,11,16-19**].

In Europe, it was Kretschmer who initially coined the term “Apallic Syndrome” for his patients who were awake but unresponsive, thereby indicating that the AS could result from either acute or chronic progressive brain lesions (Kretschmer E: Das apallischen Syndrom. Z ges Neurol Psychiat 1940, 169:576-579). Expecting a complete disconnection of the neocortical structures, Kretschmer chose the term *“apallic” *from the Latin *pallium*, translated from the old Greek word meaning in English *overcoat* in terms of *brain cortex*, explaining that functional remission can be possible. Bryan Jennett stated, quotation: “although several authors in continental Europe have used this term AS (see Dalle Ore GD, Gerstenbrand F, Lücking CH: The Apallic Syndrome; Berlin, Springer 1977), it has never caught on in English-speaking countries”, end of citation (11, p 1 end of first paragraph). Also, Jennett stressed the fact that currently AS is mainly known in the medical community as (persistent) vegetative state ((P)VS). For more details on the history of PVS and AS, different terminologies and their relevance, the clinical assessment and misdiagnosis, medical recommendations and prognosis, and last but not least ongoing critics on the term vegetative and persistent vegetative state (PVS) see [**7,9,10,11,13,14**].The term “VegetativeState” was first coined by Jennet and Plum in 1972 (Jennett B, Plum F: Persistent vegetative state after brain damage (A syndrome in search of a name. Lancet 1972, 1:734-737).They have chosen the name vegetative state to refer to the preserved vegetative nervous functioning, meaning these patients have (variably) preserved sleep-wake cycles, respiration, digestion or thermoregulation. The term “persistent”, added later, was to denote that the condition remained for at least one month after insult. In 1994, the Multi-Society Task Force on PVS defined the temporal criteria for irreversibility (i.e. more than one year for traumatic and three months for non-traumatic (anoxic) etiology) and introduced the notion of “permanent vegetative state”. In contrast to coma (which is an acute and transitory condition, lasting no more than days or weeks), the vegetative state may become chronic (lasting for decades) or may remain a transitory condition on the way to further recovery. The authoritative code of practice, published by the Royal College of Physicians in London in 1996, entitled “The permanent vegetative state”, recommended to use “VS” only for the acute condition (that could be reversible), and the term “continuing VS” when lasting more than four weeks after the insult, while “permanent” was considered to have become irreversible (by agreed criteria). It is in these latter cases that ethical and legal aspects of end-of-life issues with regard to withholding and withdrawal of life sustaining treatment are related to individuals in so-called full stage permanent vegetative state conditions [**8,20-26**].

Over the past three decades, the terminology VS, AS and its correct clinical assessment, the appropriate (clinical and scientific) diagnostic procedures, prognosis and best quality management during both the acute and chronic stages have been the subject of sustained scientific and moral-legal debates [**7,8,11,14,15**].

## Results

At present, epidemiology in Europe shows constantly increasing figures of coma survivors in Apallic Syndrome (AS) / Vegetative State (VS) because of the widespread use of advanced rescue, emergency services, and intensive care treatment with long-term artificial ventilation after acute brain damage. The impact of neurosurgical decompressive craniectomy on the prevalence of VS/AS has not yet been demonstrated, although less TBI and stroke patients present the full stage syndrome when compared with former years. In addition, the standard of activating caring and home nursing has been constantly improved for the completely dependent AS/VS “end stage” individuals, who suffer from progressive incurable neurological diseases [**3,5,10,11**]. The patients have either awakened from coma (i.e. showing eye opening, incompatible with the diagnosis of coma) or become wakeful, yet remaining clinically completely unresponsive in the end-stage of a non-treatable chronic neurological disease (they only showed reflex movements as is also the case in coma).

**Table 1:** Clinical feature of Vegetative State / Apallic Syndrome/ UWS (Modified from Gerstenbrand 1977, the Royal Collage of Physician Working Group 1996, and 15)

1	Four clinical criteria all to be fulfilled: No evidence of awareness of self or environment. No volitional response to visual, auditory, tactile or noxious stimuli. No evidence of language comprehension or expression. Cycles of eye closure and opening simulating sleep and waking (new-born like)
2	Sufficiently preserved hypothalamic and brain stem function to maintain respiration and circulation
3	The eyes are in divergent position; they can neither focus objects nor follow them; blinking reflex positive; diameter of pupils changing (enlarged-diminished) retained papillary response to light; oculo-cephalic reflex-doll-head phenomenon- partly positive.
4	No nystagmus but conjugate or dysconjugate tonic response to caloric testing. No visual fixation, tracking of moving objects with eyes or response to menace.
5	Decerebrate positioning (brain damage of internal capsule affecting the corticospinal tracts) with adducted upper limbs and flexed position of arms, palms, and fingers, and flexed-extended position of both legs, with plantar flexion of the feet and stretched position of the trunk, spastic increased muscle tonus (rigido-spasticity) Stereotype movements with lack of spontaneous and meaningful finalizing movements of face, limbs, and trunk. May be occasional movements of head and eyes towards sound movement, and trunk and limbs in purposeless way. Decerebrate body position (midbrain syndrome) spasticity with upper limbs extended in adduction and hyper pronation and the lower limbs extended with plantar flection of the feet.
6	Demonstrate primitive motor patterns such as chewing- suckling automatism (spontaneous and elicitable by stimuli), and may have oral and rasping reflexes, musculus mentalis reflexes, startle myoclonus, tonus regulating reflexes (symmetric, asymmetric neck reflex)
7	Disinhibiting of the autonomic regulating system that may provoke primitive emotional reactions and mass movements of trunk and limbs which are accompanied by vegetative reactions
8	Swallowing reflex may be preserved in most patients
9	Grimace to pain. May have roving eye movements
10	Incontinence of bladder and bowel. Spontaneous blinking and usually retained papillary and corneal responses to ice-water caloric testing; cilio-spinal reflex positive

**Table 2:** Apallic Syndrome – Modified Innsbruck Remission Scale – 8 Phases (Gerstenbrand, 2011)

Phase 1	Deep somnolent, temporary open eyes, optical fixation, sleep-wake rhythm fatigue regulated, primitive emotional reactions, primitive motor patterns partly diminished, flexed-stretched extremity position, remaining mass movements, rigido-spasticity.
Phase 2	Somnolent, optical tracking, sleep-wake rhythm begin of day time regulation, emotional reaction tendency to differentiation, primitive motor patterns tendency aim directed, diminished mass movements, tendency to finalizing, diminishing of flexed-stretched body position, diminished of rigido-spasticity.
Phase 3	Begin of responsible wakefulness, somnolence phases, following simple commands, emotional reaction differentiated (positive, negative), primitive motor patterns differentiated (higher organized grasping reflexes, oral reflexes), oral feeding accepted, first finalized movements initiated by commands.
Phase 4	Klüver-Bucy-Phase, wakeful, sleep-wake rhythm daytime regulated, object grasping with attempt to bite and chew, no recognition of the objects, increased interest for the genital region, reacting to simple orders, producing primitive sounds, beginning signs of local brain lesions, rest of flexed-stretched body position, rest of rigido-spasticity.
Phase 5	Post Klüver-Bucy-Phase, wakeful, sleep-wake rhythm day-night adapted, slight rest of flexed-stretched body position, slight signs of spasticity, diminishing of the Klüver-Bucy-patterns, increasing of finalized movements, production of simple words, upcoming of local brain lesion signs.
Phase 6	Phase of Korsakow-Symptoms, fully awake, disorientation, fully guidable fatigue phases, rest of primitive motor patterns, rest of spasticity, directed finalized movements, guidable, begin of walking.
Phase 7	Phase of the amnestic syndrome, fully awake, day-night regulated sleep-wake rhythm, rest of disorientation, fully guidable, severe memory disturbances, rest of primitive motor patterns, slight signs of spasticity, prompt finalized movements, marked diffuse and local cerebral lesions possible, symptoms of Bed Rest Syndrome.
Phase 8	Phase of the end of remission state; begin of defect state, undisturbed consciousness, normal sleep-wake rhythm, increased fatigue phases, marked symptoms of diffuse and/or local cerebral lesions (neurological, cognitive behavior deficits) possible, Bed Rest symptoms, remarks of different handicaps.

Both VS and AS full stage are characterized in the same manner by a uniform distinctive behavioural syndrome of unresponsive wakefulness (**Table 1**). This is independent of the cause affecting (a) the brain structures, (b) personal behaviour, and (c) consciousness/self-awareness [**9-11,22**]. The term *apallic syndrome* (AS) was coined by Kretschmer to describe the behavioural feature of a patient who was awake but unresponsive secondary to severe brain damage [**7,18**]. So the clinical manifestation of AS was explained as a multimodular functional disconnection syndrome of pathological neurobehavioral signs. AS patients, when awaking from coma, show the characteristic full stage feature (**Table 1**). The first (full) stage ends when the patient’s spontaneous eye movements start focussing, when eye tracking with or without turn of the head in the direction of sudden noise or movements can be observed and the patient becomes regularly able to follow reproducible simple commands in a predicting manner (a motor response, even if shaky, nevertheless stands as a very important initial event) [**7,11,17,22**].

In this respect, the differential diagnosis of the early remission stages is mandatory. Therefore, the Aspen Neurobehavioral Conference Workgroup (von Wild and Gerstenbrand took part on invitation in the Seville meeting in 1996) characterized a new clinical entity and coined the term ‘minimally conscious state’ (MCS) (**Table 3**), describing patients who have recovered from a vegetative state (meaning they show more than reflex motor behavior but fail to show functional communication or object use) [**23**]. MCS turned out to be clinically most helpful and reliable in differentiating the unresponsive wakefulness of full stage VS/AS syndrome from the earliest remission stages. MCS describes evidence of limited but clearly discernible self or environmental awareness on a reproducible or sustained basis by neurobehavioral criteria. Katz reported a prevalence of MCSin the US eight times higher than VS [**24**].

**Table 3:** Minimally Conscious State (MCS) Criteria (Giacino JT, Ashwal S, Childs N, Cranford R, Jennett B, Katz DI, Kelly JP, Rosenberg JH, Whyte J, Zafonte RD, Zasler ND. Neurology. 2002;58(3):349-53 2, 349-353, 2002)

Definition: Evidence of limited but clearly discernible self or environmental awareness on a reproducible or sustained basis, by one or more of these behaviors:
1	Simple command following
2	Gestural or verbal “yes/no” responses (regardless of accuracy)
3	Intelligible verbalization
4	Purposeful behavior including movements or affective behaviors in contingent relation to relevant stimuli; examples include:
a	appropriate smiling or crying to relevant visual or linguistic stimuli
b	response to linguistic content of questions by vocalization or gesture
c	reaching for objects in appropriate direction and location
d	touching or holding objects by accommodating to size and shape
e	sustained visual fixation or tracking as response to moving stimuli

**Table 4:** Aetiology of the Vegetative State/Apallic Syndrome (7-11)

1.1	acute traumatic :secondary to acute traumatic brain injury (TBI)
1.2	acute non-traumatic: secondary to hypoxic-ischemic encephalopathy because of intracranial space occupying lesions, cardio respiratory arrest ( i.e. sudden infant death syndrome), perinatal asphyxia, strangulation, cerebrovascular (i.e. cerebral haemorrhage, cerebral infarction, subarachnoid haemorrhage), metabolic disorders (i.e. hypoglycaemia, hyperglycaemia, uraemia, hepatic, thyreotoxic, others), intoxication (i.e. endogen and exogenous CO, drug poisoning, mercury, snake venom (viper), plant, animal poisons etc.)
1.3	chronic inflammatory, degenerative, metabolic (including intoxication) as the final stage of progressive diffuse or multilocular brain lesions for example Creutzfeld-Jacob disease CJD), Alzheimer’s disease (AD), Pick’s disease, Huntington’s chorea, severest forms of multiple sclerosis, adrenoleukodystrophy (Flatau-Schilder disease), Marchiafava-Bignami disease, chronic hepatic failure, Minamata disease.
1.4	Developmental malformations as they are anencephaly, hydranencephaly, Lissencephaly, holoprosencephalyencephalocels, schizencephaly congenital (decorticate) hydrocephalus, severe microcephaly

Concerning the etiology (**Table 4**) we would like to refer to the literature [**7,11**]. In Germany, the ICD code is G 93.80 for VS/ AS treatment, which does not reflect the severity of brain damage and etiology. The cause of VS/ AS is coded by G 93.1 for hypoxic brain lesion, S 06.20 for diffuse brain damage, I 61.6 intracerebral mass hemorrhage, G 30.9 Mb Alzheimer. In case AS G 93.80 is in combination with the code of procedures 8-552 for early neurorehabilitation, this VS/AS patient will be assessed as B 43Z, and no costs will be refunded for the first two weeks of clinical treatment.Thereafter, from day 14 to 28, a flat rate will be paid including all treatment costs. However, in case the VS/AS patient stays longer than 28 days in the early rehabilitation unit, the individually assessed local daily compensation will be paid for the patient, covering all expenses of in-hospital treatment from the first day.

The careful neurobehavioral assessment by clinically experienced specialists in VS/AS ranks first in the diagnostics (**Table 1**) as was previously stressed already in our EFNS task force consensus on best practice [**7**]. The VS/AS full stage is characterized by the classical symptoms and signs of wakefulness without awareness of self and environment and related signs and symptoms of functional disinhibition of upper brainstem. Evaluation needs a considerable amount of time, measured in weeks rather than hours and during different day times, if varying levels of function are to be identified and correctly classified on a special questionnaire. The ability to generate a behavioral response fluctuates from day to day, hour to hour, and even from minute to minute. Sampling and documentation of spontaneous behavior as observed by the nursing staff and family members [**3,7,10,22,25,26**] is necessary.

Somatosensory evoked potentials (SSEPs) have been proposed as acceptable predictor of output in the early phase of coma and AS with a higher predictive value above 90% compared to EEG reactivity in two thirds comparable to GCS (class IIevidence) (148). Median nerve SSEPs correlate with outcome inhypoxic AS cases (Binder; Saltuari, unpublished data). Laureys et al, in a Positron Emission Tomography (PET) study on patients in full stage AS, recorded a cascade of functional disconnections of auditory evoked potentials along the auditory cortical pathway from the primary auditory area to parietal associative and limbic areas, when the primary auditory cortex was activated by appropriate stimulation and a brain stem response was recorded [**27**]. Kotchoubey et al [**28,29**] reported the observation of P300 responses in 14 AS patients and 19 cases of minimal responsiveness. Laboratory data suggest possible clinical use of P300 as an index of brain activity during recovery from AS for the future. Schoenle et al [**16**] tested the existence of cognitive capabilities in AS by using the N-400 ERPs paradigm and reported significantly more intact semantic capabilities (=76.7%) for brain injured “near AS” than AS patients (12%).

**Figure 1:**
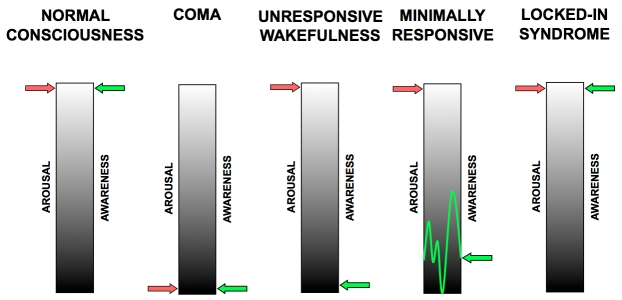
Impairment of consciousness (compare Prigatano, 32) (Sketched by Steven Laureys, 2004)

Cranial computerized tomography (CCT) and magnetic resonance imaging (MRI) have become the gold standard, when available. CT and MRI have proved to be of high diagnostic sensitivity but without any AS specificity for demonstrating brain pathologies, i.e. brain atrophy and diffuse axonal injury (DAI) secondary to shearing injuries. Primarily bilateral brain lesions within the upper brainstem, pons and mesencephalon in early MRI imaging have shown to be of significant prognostic value. Functional MRI studies have demonstrated a residual capacity in minimally conscious patients to activate large integrative networks. Recent data from Laurey’s laboratory [**30,31**] did not allow differentiating AS from minimally conscious patients (MCP) by overall decreased cerebral metabolism, but fMRI studies with the aid of a simple auditory activation paradigm made this possible, when each and every of five MSP demonstrated a more widespread activation than did any of the 15 AS patients.

**Figure 2.1:**
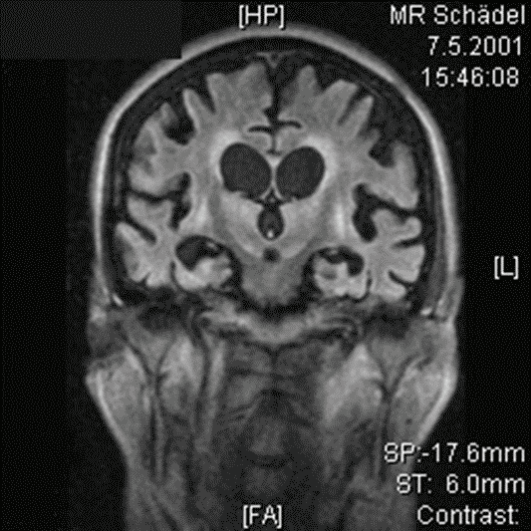
FDG PET scan PVS full stage. Indication: Brain metabolism 10 months after insult. L.D., female, 66 years: PVS full stage following intraoperative cardiac arrest September 2001. Despite immediate resuscitation severe cerebral hypoxemia, massive dehydratation and electrolyte imbalance; postoperative coma. Begin of early neurosurgical rehabilitation 2001/04/25. MRT of the brain (2001.05.07): Massive cortical and subcortical brain atrophy with dilated ventricles (normal intracranial pressure) following cardiac arrest and resuscitation.

**Figure 2.2:**
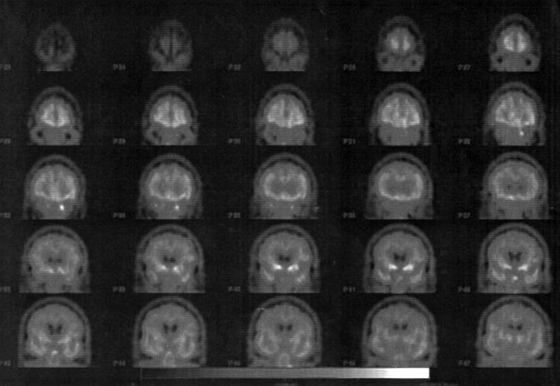
FDG PET scan PVS full stage. Indication: Brain metabolism 10 months after insult. L.D., female, 66 years: PVS full stage following intraoperative cardiac arrest September 2001. Despite immediate resuscitation severe cerebral hypoxemia, massive dehydratation and electrolyte imbalance; postoperative coma. Begin of early neurosurgical rehabilitation 2001/04/25. (black-white) FDG-PET of the brain FDG PETscan six months after the insult shows no pharmacological stimulation of the cortical and subcortical brain structures after intravenous application of Amantadine® (an NMDA-antagonist that blocks N-Methyl-D-Aspartat-receptors in the brain and so glutamate). rCBF was less than 20ml/ 100gr/minute.

Recent PET studies [**33**] and fMRI examinations (Gerstenbrand et al., work in progress) of unresponsive wakeful patients have been of help to differentiate the UWS from Locked-in Syndrome. PET and single-photon emission computed tomography (SPECT) studies have demonstrated the impairment and pathological distribution of cerebral blood flow (CBF), when in VS/AS the cerebral metabolism is reduced to about 40-50% of normal values. Pathophysiology has shown that cerebral coma can be assumed at a persistent flow rate below 20mL/ 100g/min, which provokes definitive cortical cell loss and atrophy. Reduction of CBF mainly in the basal ganglia and mesencephalon is a characteristic feature [**30**]. In addition to pharmaco-EEG studies, we recommend central activating drug PET and/or SPECT studies for selected cases. We used these methods, such as Fluor-Glucose–PET studies, before and after pharmacological stimulation. Amantadine and Bromocriptine, for example, can demonstrate the extent of functional metabolic brain damage on CBF in the full and remission stages [**7**]. 

Recent PET studies have indicated that some AS patients are unconscious not just because of a global loss of neuronal function, but due to an altered activity in a critical fronto-parietal cortical network and abolished functional connections within this network and with non-specific thalamic nuclei.

Recovery of consciousness (Figure 1) is dependent on the restoration of this cortico-thalamo-cortical interaction [**15,32**]. Kanno et al used CBF and PET studies for intravenous drug stimulation tests to activate brain metabolism of comatose and VS patients secondary to hypoxic brain damage (**Figures2and3**). FES spinal cord stimulation seems only indicated in case CBF is >20mL/100g/min and PET or SPECT show an increase of brain metabolism after stimulation [**7**]. 

Up to now, there are no clear prognostic criteria whatsoever for the functional improvement along with the characteristic signs and symptoms of the VS/AS remission phase as differentiated by Gerstenbrand (**Table 1**) and / or extended GOS. Patients could frequently pass the initial full stage (acute midbrain syndrome) and reach higher cortical improvement over time, if it is secondary to acute brain damage (about 80%) while following hypoxemia, chances are less good.

**Figure 3.1:**
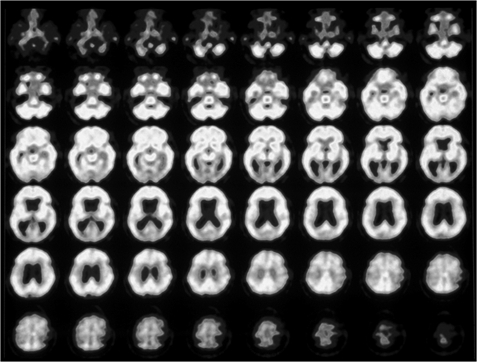
Pharmaco- D2O18 Fluor- Glucose PET in PVS Indication: Final decision on necessity for further early neurosurgical rehabilitation (German Coma Remission Scale 9 points equivalent PVS/AS) 10 months after the insult.(D.L., f, 66 ys – PVS full stage 11 months after acute hypoxia due to cardiac arrest during minor gynecological intervention with immediate resuscitation, same patient as **Figure 1**). (black-white) D2 O18 Fluor-Glucose–PET Study of the brain in PVS full stage (2001.06.28).

**Figure 3.2:**
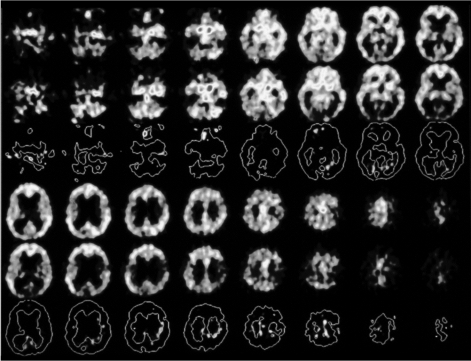
Pharmaco-(Amantadine sulfate-) D2 O18 Fluor- Glucose PET in PVS. Upper half of the horizontal brain scans before the application of PK Merz ®, lower half of the brain sections after intravenous application of pKMerz® (Amantadine sulfate). No differences of the regional cerebral blood flow (rCBF), not pharmacological stimulation of severely decreased brain metabolism within the cortical and subcortical brain structures.

There is a need for categorisation, based on regular assessment, to define whether a patient is actually progressing from a functional perspective and to identify those who are no longer apallic, which has to be stated in evidence on a special questionnaire form as part of the charts [**25**]. In case of uncertainty, this can be called borderline or transition stage. Recovery from AS is defined as the ability to establish visual and/or oral contact with the outer world and to reproducibly obey meaningful commands. During functional remission, stage 8 phases can be recognized (**Table 3**) when phase 3 to 5 mimic typical symptoms of the Klüver-Bucy syndrome, promising a good prognosis [**7-10**]. Restoration of higher cerebral functioning is due to its (preserved) brain plasticity (Stein), which can be facilitated and supported by rehabilitative interventions over the time course, depending on the patient’s age, otherwise physical condition, and the primary cause. Holistic rehabilitation aims at the AS patient’s final social reintegration. The International Working Party on the Management of AS described three possible main patterns of clinical recovery from full stage AS to mental–cognitive awareness: 1. a continuum through a series of levels or phases as previously described by Gerstenbrand; 2. a number of discrete syndromes with specific patterns of recovery; 3. a branching tree along which different sub-categories of patients may pass.

The first full stage (**Table 1**) ends and remission starts (**Tables2and3**) at the time when a) the patient’s spontaneous eye movements start focussing, b) eye tracking with or without turn of the head in the direction of sudden noise or movements can be observed, c) the patient becomes regularly able to follow reproducible simple commands in a predicting manner (a motor response, even if shaky, nevertheless stands as a very important initial event). Unfortunately, there are only three evidence based class II analyses reported with regard to quality management and outcome of apallic patients (full stage and remission stage) after one year. The latter epidemiological studies give reference to the new classification of ICD code 780.03. In the prospective [**10**] and the prospectively controlled and population based studies by Stepan/Binder (3,25) and our study on TBI [**7**] in the Vienna region, Austria (1,620,170 inhabitants in 2001), 9% out of 78 patients had a mistaken diagnosis of AS [**22**], cross-checked against retrospectively collected data from 1996 to 2000, (ICD-9 diagnosis). In Germany, in 2000/2001 [**7**], there were three AS adults both at the end of early rehabilitation and after one year out of 6,782 acute TBI of all severities (ICD-10 criteria), which corresponds to 2% of 175 rehabilitations. Therefore, the prevalence after (severe) TBI was 0.13/ 100,000 in the year 2001 for adults; no children, no mild or moderate TBI suffering from VS/AS assessed by initial GCS and outcome GOS

**Table 5:** PVS as part of the Glasgow Outcome Scale Score (GOS) Impact of initial GCS on TBI outcome at discharge from in-hospital neurorehabilitation (N = 258)

GOS	GCS at the Beginning				
	mild	moderate	severe	no data	number
5 no/minimal functional deficits	46	8	-	15	69 26,7 %
4 moderate disability	40	12	3	8	63 24,3 %
3 severe disability	15	13	10	-	38 14,7 %
2 vegetative state VS	-	-	3	-	3 1,2 %
1 dead	-	1	1	1	3 1.2%
missing	14	7	2	59	82 31,8 %
total number %	115 100,0 %	41 100,0 %	19 100,0 %	83 100,0 %	258 100,0 %

**Table 6:** GOS changes of functional outcome following neurorehabilitation within the first year (for GOS see Jennett B, Bond M. Assessment of outcome after severe brain damage. Lancet. 1975;480—484)

From the prospective controlled TBI study in adults, showing an on-going, age dependent restoration of cerebral functioning due to and after neurorehabilitation within the first year (7).
GOS (% of treated adult TBI) at the end of Early Neurorehabilitation (N=75)	
1 = Dead	4
2 = VPS	2,7
3 = Severedisability	37,3
4 = Moderate disability	27
5 = Minor disability / goodrecovery	29
GOS (% of all rehabilitated adult TBI) at one year after brain damage (N=180)	
1 = Dead	1,2
2 = VPS	1,2
3 = Severedisability	22,6
4 = Moderate disability	36
5 = Minor disability / goodrecovery	39

Concerning the ongoing debate as raised by the nomenclature, Jennett himself [**11**] stressed the fact that AS is mainly known and used in continental Europe, while in English speaking countries the medical community uses (persistent) vegetative state ((P)VS). Jennet and Plum first coined this term in 1972 (Jennett B, Plum F's famous paper: Persistent vegetative state after brain damage. A syndrome in search of a name. Lancet 1972, 1:734-737). The name "vegetative state" was chosen to refer to the preserved vegetative nervous functioning, meaning these patients have (variably) preserved sleep-wake cycles, respiration, digestion or thermoregulation. In contrast to coma (which is an acute and transitory condition, lasting no more than days or weeks), a vegetative state may become chronic (lasting for decades) or may remain a transitory condition on the way to further recovery. The adjunct *persistent*, then *permanent*, and lastly *continuing* was added over the years to denote that the condition might remain unchanged from one month after insult up to one year and more. In 1994, the Multi-Society Task Force on PVS defined the temporal criteria for irreversibility (that is, more than one year for traumatic and three months for non-traumatic (anoxic) etiology) and introduced the notion of *permanent* vegetative state. The authoritative code of practice, published by the Royal College of Physicians in London in 1996 entitled ”The permanent vegetative state”, recommended to use VS only for the acute condition (that could be reversible) and the term *continuing* VS when lasting more than four weeks after the insult, while *permanent* was considered to have become irreversible (by agreed criteria). It is owing to these latter cases that in recent years a controversial discussion started about the ambiguity of the persistent component of PVS when it might suggest irreversibility, which in turn can result in suboptimal or even no early neurorehabilitation efforts at a stage when all rehabilitative measures are important to restore higher cortical functioning, awareness and cognition. Different parties, including the Pro-Life-Committee of the Catholic Bishops in the US and a personal address of Pope John Paul II “on life-sustaining treatments and vegetative state: scientific advances and ethical dilemmas" [**24**], have expressed deep concern about the label PVS when the word "vegetative" suggests that the suffering patient is more like a vegetable than a human being. This is the reason why ethical and legal aspects have become so important for lawyers, philosophers, and others outside medicine in the discussions over end-of-life issues with regard to withholding and withdrawal of life sustaining treatment (that is, antibiotics, artificial hydration and nutrition are related to individuals in so-called full stage persistent vegetative state conditions). Even today, after four decades, we can say, “The vegetative state - A syndrome in search of a name” [**11**]. So, in 2009, the European Task Force on Disorders of Consciousness, having in mind the worldwide concern regarding the negative connotation inherent in the term "vegetative state" and its possible effect on vulnerable patients awakening from coma, who sometimes never recover any voluntary responsiveness but may (probably more often than initially believed) recover minimal signs of consciousness, proposed to change the term "vegetative state" itself to "Unresponsive Wakefulness Syndrome" or UWS [**15**]. Now physicians can choose this neutral descriptive term to refer to patients, who, as the term indicates, show a number of clinical signs (hence the use of syndrome) of unresponsiveness (meaning they fail to show non-reflex behavior or command following) in the presence of wakefulness (meaning they open their eyes spontaneously or upon stimulation). Given the above-mentioned difficulty in making strong general claims about awareness in severely brain damaged patients, we have chosen to use the clinically descriptive term "unresponsive" rather than the misleading "unaware".

## Discussions

The term "apallic" cannot be explained by or taken nowadays for a conditio sine qua non of an anatomically completed and permanent disconnection of neocortical structures and of higher cerebral functioning from the midbrain when, awaking from coma, functional remission is possible to a certain stage (**Table 2**) as opposed to the individuals suffering from final end stage of an incurable progressive neurological disease. In so far “apallic” is functional-anatomically incorrect, but clinically still appropriate for neurobehavioral symptoms, signs, and functional remission. Bycontrast, persistent “vegetative” state (PVS) must not necessarily be “persistent“, why Jennett changed the phraseto "permanent", well aware that one has to accept that the abbreviation “P” will still widely be used to mean persistent rather than permanent in a non-acceptable misleading way, albeit a persisting negative connotation of "vegetative" [**11**]. The name “vegetative” was originally chosen to refer to the preserved vegetative (autonomous) nervous functioning of preserved sleep-wake cycles, respiration, digestion and thermoregulation. We shared the concerns of others and the on-going controversy over the meaning of “PVS” nomenclature (Jennett, personal communication 1996; **4,7,8,10,13-15,32**) so that Jennett [**11**] finally dropped the attribute “P” for persistent and/or permanent. 

In reviewing the literature and by our own clinical and scientific experience over more than half a century, we strongly recommend herewith to all physicians, neuropsychologists, social health care authorities, and politicians to disclaim the term PVS and all the other misleading terminologies and use instead in the future only Unresponsive Wakefulness Syndrome (UWS) as new name.

## Conclusion

In line with an intensive critical analysis of the conceptual VS and AS domains in the light of recent results in research, we would like to suggest herewith that the new and neutral, humane and well descriptive name of *Unresponsive Wakefulness Syndrome*, abbreviation UWSbe adopted instead. This will prove to be a practical and reliable alternative to "vegetative state" or "apallic syndrome", which we view as outdated. It was Jennet who expressed the concern that the term "PVS" still continues to have strong negative connotations after more than 40 years of use (routinely in GOS assessment), inadvertently risking comparisons between patients and vegetables and implying persistency from the moment of diagnosis. UWS will help the medical community and related public to finalize the discussions. UWS is a clinical syndrome-describing patients who fail to show voluntary motor responsiveness in the presence of eyes-open wakefulness.

### Addendum [24-26, 33-42]

In the light of the ongoing controversial and just recently intensified discussions in the medical community and public on the individual’s human rights and doctor’s responsibility regarding end-of-life decisions, it seems worth recalling here some of our concerns and suggestions with reference to new arguments. Firstly, the United Nations *Standard Rules on the Equalization of Opportunities for Persons with Disabilities* are not yet followed by all states, politicians, and societies because of different distinctive features of social-economic and cultural-religious capacities. The Convention marks a "paradigm shift" in attitudes and approaches to persons with disabilities from viewing persons with disabilities as "objects" of charity, medical treatment, and social protection towards viewing persons with disabilities as "subjects" with rights, who are capable of claiming those rights and making decisions for their lives based on their free and informed consent as well as being active members of society. In contrast to the former decision in 2006, when medically assisted suicide was taken as prosecuted criminal act, the Federal High Court of Justice in 2010 changed the law towards the advanced directive – the individual's last will. Therefore, the German Federal Medical Association finally agreed to accept medically assisted suicide on the premise of correctly formulated last will decisions by the person having legal capacity. This looks like opening the door for active euthanasia in Germany. In the Netherlands, Belgium and Luxemburg medicine is exempt from punishment when strict legal regulations are followed; in Switzerland, medically assisted suicide is permitted while euthanasia, like in France, Italy and in Germany, remains a prosecuted criminal act.

In Romania, treatment is part of the patient’s legally stated rights, as long as it is not possible to know the patient’s personal decision (patient with altered consciousness state), there is no deliberately written expression of his/ her refusal of any medical intervention (being informed about the consequences) or his/ her solicitation to stop it, voluntarily expressed in written form, which is laid down in the Law No. 95/ 2006 (article 376), regarding the Reform in the Sanitary Domain - with further modifications and completions [**38**] and in the Law No. 46/ 2003 (Article 13, 16) of the patient’s rights [**39**] following our Medical Deontology Code [**40**]. The Medical Deontology Code stipulates that “The incurable patient will be treated with the same care and attention as those patients likely to be cured”; the physician (medical doctor) will act/ continue to act to the best of his/ her knowledge, skills and material resources, to assist and treat as long as the patient is alive, including those in vegetative states, or until their kin/ legal carers solicit the patient’s discharge. Moreover, even if such a request is made in written form by the patient’s legal representative, this has to be in consensus with the physician (medical doctor)’s opinion in accordance with article 17, paragraphs 1 and 2 of the Law No. 46/ 2003 of the patient’s rights: (1) In case the medical service providers consider the intervention to be (still - in such cases - our note) to the patient’s benefit, and the legal representative of the patient refuses to give his/ her consent, the decision is conferred to a specialty arbitration commission. (2) The arbitration commission consists of three physicians (medical doctors) for hospitalized patients and two physicians (medical doctors) for ambulatory patients (outpatients). Hence, also in Romania euthanasia is forbidden [**40**], being considered a prosecuted criminal act - Article 175 in the Penal Code [**41**]. With total good will and the best knowledge, skills, and available material resources the patient’s interest represents the supreme ethical commitment and obligation for any physician.

In Italy, for example, we became involved in the case of *Eluana Englaro*, 38 years old, to prevent the withdrawal of nutrition and fluid as legally permitted in the sense of a suspected last will of the person having been diagnosed to be in permanent vegetative statefor 17 years, but who was obviously minimally conscious and reactive. Even officials from the Catholic Church and the President of Italy, *Silvio Berlusconi*, entered into the case (41 – becomes 42). This reminds us of *Terri Schiavo*, US. Quotation from the internet (42 – becomes 43), when President *G.W. Bush* took over the case, saying: “Executive summary: Human vegetable, 1990-2005. *Terri Schiavo*, born 1963, entered a vegetative state in 1990 after adopting an "iced tea diet" (related to her bulimia) that had resulted in a disastrous potassium deficiency, which caused irreversible brain damage. Politicians inserted themselves into the fray. The case was the catalyst for Florida's controversial "Terri's Law", which gave Governor *Gorge W. Bush* the authority to have *Schiavo*'s feeding tube re-inserted when a court ruled that her husband could have it removed. As the insanity moved to the federal level, Schiavo's feeding tube was finally removed on March 18, 2005, and her heart stopped beating 13 days later" end of quotation.

If there is clinically no doubt regarding the diagnosis of UWS, and given the findings that repeated assessments unequivocally demonstrate the irreversibility of signs and symptoms of the pathological *full stage* behavioural syndrome – notwithstanding the best possible intensive care therapy and early rehabilitation measures over more than six months in the case of hypoxic AS, and more than twelve months in the case of posttraumatic AS – then, under consideration of the specific facts of the individual case, the question can be raised regarding the further active therapy of the patient and the issues of artificial nutrition and liquid intake via a PEG probe. This decision must be based on the concurrent diagnoses of two independent neurologists and/or neurosurgeons with proven expertise in the field of neurological intensive care medicine and the treatment of AS patients. How to prevent frequent misdiagnosis VS/ AS/ UWS is mentioned and discussed in our paper [**7,10**]. Only on the basis of strict diagnostic criteria and the written last will might it be permissible to end all life prolonging medical measures – or, more correctly, measures that prolong the patient’s sufferings – and thus enable the unresponsive, but wakeful and irreversibly severely brain damaged patient to die in a way compatible with his rights as a human being. Major ethical objections against the ending of artificial nutrition upon medical advice and approval should be interpreted as arguments for the value of human life. All severely disabled individuals have the same dignity and right to live like a person who is healthy from the mental-cognitive viewpoint. Devoted care is an expression of human solidarity, and the helpless VS/ AS / UWS patient is existentially reliant on dedicated protection and the appropriate degree of care by society. The enlightened person of this day and age rightfully expects that the physician in charge of his treatment feels committed to both the indivisible value of life as such, but also to respecting the patient’s will (witnessed and confirmed patient’s declaration). The patient’s right to self-determination is binding for all concerned and must be respected by the physician even in cases when this will does not coincide with his (expert) medical opinion. In most European countries, active euthanasia is subject to legal sanctions and is classified as criminal act. Palliative medicine, by contrast, is considered an act of mercy through the physician in the form of withholding treatment. This attitude does not relieve the physician from his obligation to continue to provide the patient with conscientious care and sufficient palliative, i.e. pain relieving, therapy on top of human attention. 

This, however, prompts the community to reflect the *Research Involving Human Subjects* in the Helsinki Declaration of 1964 and its numerous amendments, when the doctor of today is obliged to devote himself to using all available means to help his patient and to leave nothing untried. 

Paragraph 10: Physicians should consider the ethical, legal and regulatory norms and standards for research involving human subjects in their own countries as well as applicable international norms and standards. No national or international ethical, legal or regulatory requirement should reduce or eliminate any of the protections for research subjects set forth in this Declaration.

Paragraph 35: In the treatment of a patient, where proven interventions do not exist or have been ineffective, the physician, after seeking expert advice, with informed consent from the patient or a legally authorized representative, may use an unproven intervention if in the physician's judgment it offers hope of saving life, re-establishing health or alleviating suffering. Where possible, this intervention should be made the object of research, designed to evaluate its safety and efficacy. In all cases, new information should be recorded and, where appropriate, made publicly available.

### Acknowledgements

Sources of partial funding Laureys et al. BMC Medicine 2010, 8:68 http://www.biomedcentral.com/1741-7015/8/68) originated from the European Task Force on Disorders of Consciousness, founded by G. Dolce, following the meeting in Rome at the Italian Ministry of Health, Work and Welfare, 18 September 2009, funded by the S. Anna Institute, Crotone, Italy. Over many years, early neurosurgical rehabilitation, educational training of the team, and scientific research on guidelines on AS quality management of K. von Wild were granted by Cerebprotect association for the development of early neurorehabilitation e.V., Muenster, D.
